# Abdominal surgical‐site *Fusobacterium nucleatum* pyomyositis in an immunocompetent adolescent

**DOI:** 10.1002/kjm2.12562

**Published:** 2022-05-24

**Authors:** Chao‐Ting Tai, Min‐Sheng Lee, Yu‐Tang Chang

**Affiliations:** ^1^ Department of Surgery Kaohsiung Medical University Hospital Kaohsiung Taiwan; ^2^ Division of Pediatric Infectious Disease, Department of Pediatrics Kaohsiung Medical University Hospital Kaohsiung Taiwan; ^3^ Division of Pediatric Surgery, Department of Surgery Kaohsiung Medical University Hospital Kaohsiung Taiwan; ^4^ Faculty of Medical School, College of Medicine Kaohsiung Medical University Kaohsiung Taiwan

A 16‐year‐old male patient presented to the emergency department with progressively worsening right lower abdominal pain for 2 weeks. He had had an appendectomy 3 years previously. Vital signs were stable with physical examination disclosing tenderness and guarding over the right lower abdomen. Lab data revealed leukocytosis (22,710/μl) with neutrophils predominant (84.4%) and elevated C‐reactive protein (63.93 mg/L). Enhanced abdominal computed tomography (CT) scan revealed a lobulated abscess at the right lower abdominal wall with increased peripheral infiltration (Figure 1), a finding consistent with the diagnosis of pyomyositis. Treatment with broad‐spectrum antibiotic agents was initiated and the abscess was drained under CT guidance.

**FIGURE 1:**
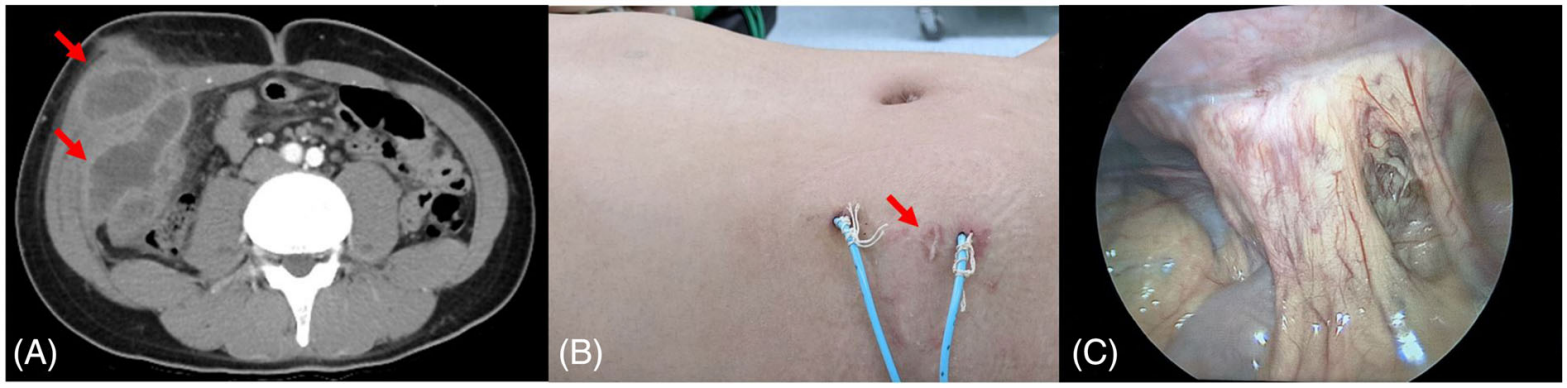
(A) Lobulated abscess at right lower abdominal wall peripheral increased infiltration (red arrows). (B) Note two pigtail catheters and previous operative scar (red arrow) at right lower abdomen, which is compatible with the location of pyomyositis. (C) Laparoscopy showing severe adhesion of the omentum to the right lower quadrant of peritoneum.

Because of continuing fever and abdominal pain, surgical drainage was later performed with simultaneous laparoscopic examination showing no intraperitoneal spillage of abscess, but severe adhesion of omentum to the peritoneum over the right lower quadrant of the abdomen was found (Figure 1). Adhesiolysis was then performed to make sure no abscess was encapsulated, with tissue submitted for pathology consisting of marked hemorrhage and infiltration of suppurative cells showing the pus culture growing *Fusobacterium nucleatum*. Following the treatment, the patient recovered without further sequelae.

Pyomyositis is a purulent infection of skeletal muscle and occurs most often in the lower extremity.^1^ Most patients with tropical pyomyositis are otherwise healthy without underlying comorbidities, while most patients in temperate regions are immunocompromised or have other serious underlying conditions.^1^, ^2^ Our case was a healthy, immunocompetent teenager (C3: 116 mg/dl, C4: 29 mg/dl, IgG: 1230 mg/dl, IgA: 472 mg/dl) with no known hematologic malignancy. Previous abdominal surgery would have caused injury to the abdominal muscle, which might have created a locus minoris resistentiae (Figure 1). As a result, the weakened muscle could then offer little resistance to microorganisms.

The cause of pyomyositis is generally regarded as the result of hematogenous spread of infection from an occult source.^2^ In our case, the blood cultures showed no bacterial growth, but the pus cultures grew *F. nucleatum*, which is a commensal flora in the human oral cavity and plays an important role in oral hygiene and periodontal disease.^3^ This patient had undergone dental caries treatment 2 months earlier, so it is reasonable to hypothesize that the hematogenous spread of *F. nucleatum* was due to weakening of the abdominal muscles resulting from previous abdominal surgery as the possible cause of infection. This also emphasizes the importance of processing specimens of pyomyositis for anaerobic bacteria, especially in patients having undergone dental procedures or experienced oropharyngeal infection previously.

The findings from this case suggest that even in an immunocompetent or no‐known‐underlying‐disease condition, surgical sites might become more prone to infection and may predispose the patient to pyomyositis. It is important for the physicians to recognize surgical‐site pyomyositis as a postoperative complication in immunocompetent patients.

## CONFLICT OF INTEREST

The authors declare no conflict of interest.
